# TMFoldRec: a statistical potential-based transmembrane protein fold recognition tool

**DOI:** 10.1186/s12859-015-0638-5

**Published:** 2015-06-30

**Authors:** Dániel Kozma, Gábor E. Tusnády

**Affiliations:** “Momentum” Membrane Protein Bioinformatics Research Group, Institute of Enzymology, Research Centre for Natural Sciences, Hungarian Academy of Sciences, PO Box 7, , H 1518 Budapest, Hungary

**Keywords:** Transmembrane protein, Statistical potential, Fold recognition, Threading

## Abstract

**Background:**

Transmembrane proteins (TMPs) are the key components of signal transduction, cell-cell adhesion and energy and material transport into and out from the cells. For the deep understanding of these processes, structure determination of transmembrane proteins is indispensable. However, due to technical difficulties, only a few transmembrane protein structures have been determined experimentally. Large-scale genomic sequencing provides increasing amounts of sequence information on the proteins and whole proteomes of living organisms resulting in the challenge of bioinformatics; how the structural information should be gained from a sequence.

**Results:**

Here, we present a novel method, TMFoldRec, for fold prediction of membrane segments in transmembrane proteins. TMFoldRec based on statistical potentials was tested on a benchmark set containing 124 TMP chains from the PDBTM database. Using a 10-fold jackknife method, the native folds were correctly identified in 77 % of the cases. This accuracy overcomes the state-of-the-art methods. In addition, a key feature of TMFoldRec algorithm is the ability to estimate the reliability of the prediction and to decide with an accuracy of 70 %, whether the obtained, lowest energy structure is the native one.

**Conclusion:**

These results imply that the membrane embedded parts of TMPs dictate the TM structures rather than the soluble parts. Moreover, predictions with reliability scores make in this way our algorithm applicable for proteome-wide analyses.

**Availability:**

The program is available upon request for academic use.

**Electronic supplementary material:**

The online version of this article (doi:10.1186/s12859-015-0638-5) contains supplementary material, which is available to authorized users.

## Background

Transmembrane proteins (TMPs) have several functions in living cells; they participate in e.g. energy production, regulation, metabolism, signal transduction and cell-cell adhesion. Consequently, many diseases can be connected with the mutations of TMPs causing dysfunction of e.g. ATP binding cassette (ABC) transporters [1–3], solute carrier family proteins (SLCs) [4, 5], various ion channels [6, 7] or GPCRs [8, 9]. For a detailed review on how non-synonymous mutations can disturb helix-helix interactions, the folding of TMPs, or their function leading to diseases, the reader is referred to ref. [10]. The pharmaceutical significance of TMPs is evident, since they cover the two third of known druggable targets [11]. Although, the biological importance of TMPs has been already realized, only a few hundred structures are determined. Compared to globular proteins, it is a negligible value. This fact is due to the special physical-chemical properties of TMPs, which make the structure determination much more difficult. Consequently, there is a huge difference between the number of known 3D structures and that of the sequences. While the average ratio of TMPs is about 25-30 % in the proteomes, they are represented with less than 2 % in Protein Data Bank (PDB). Therefore, the research of TMPs and their modeling is necessary and not only from theoretical point of view. It has also practical importance, since computer-aided drug design methods are based on 3D structure models of target proteins [12].

There are three main types of approaches to predict the structure of TMPs, namely homology modeling, *de novo* modeling and fold recognition (threading). The most widely used approach is the so-called homology modeling (mainly of globular proteins). Programs (e.g. MODELLER [13, 14], MolIDE [15]) and automatized webservers (e.g. SWISS-MODEL [16]) apply this technique to model the 3D structure of globular proteins. With prudent and careful usage, this method supplies the most reliable models. Unfortunately, homology modeling is heavily limited by the number of available sequence homologues with known 3D structures. This limitation can be overcome by recently developed HMM-based sequence search algorithms (e.g. HHBlits [17, 18]), which can correctly identify sequence homologues with less than even 30 % of sequence identity. However, this threshold constrains the usability.

*De novo* methods do not require any additional structural information for TMP structure prediction, but their performance is limited by the huge conformational search space. Although TMPs have significantly less possible conformations than globular proteins with same length due to the constraints imposed by the lipid bilayer, the average length of TMPs is greater than the globular ones. These two effects keep the search space size in the same order of magnitude as it is in the case of globular proteins. *De novo* methods can be applied mainly for short proteins [19], since, by the increasing protein lengths, the exhaustive sampling of the conformational space becomes fundamentally unfeasible [20]. Obviously, additional information, such as predicted contacts [21] or structure fragments [22, 23] can be also taken into account by the calculation, but these cannot reduce significantly the size of the search space as well as the computational time. Furthermore, these additional inputs can lead to discrepancies [24]. RosettaMembrane [25] and FILM3 [23] use structural fragments, but whole secondary structure elements can be also used to reduce the conformational space [26]. Even if *de novo* methods do not have any computational limitations, in practice, these methods provide the most ambiguous results compared to homology modeling and threading.

Fold recognition (threading) algorithms can overcome the problems arising from the lack of sequence homologues and the mapping of huge conformational spaces. On the one hand, fold recognition methods are not restricted by the need of sequence homologues. On the other hand, they provide a faster prediction and generally more reliable results than the *de novo* methods. However, regarding TMPs, there are also several difficulties of threading methods. The most important one is that the vast majority of TMP folds are still unknown. Up to now, only about one fifth of transmembrane folds has been determined [27]. It is important to note, it does not mean that only the one fifth of known sequences can be assigned to its native structure. Due to the exponential fall-off of the population of fold classes, there are only a few but highly populated classes which are already known [27]. Moreover, the number of determined TMP folds is increasing from year to year. These two factors make fold recognition more viable.

Several algorithms have been developed so far for fold recognition, but only a few of them are transmembrane-specific. The sequence-based homology detection algorithms, such as HHalign [17] and Jackhammer [28], TMFR [29] and threading methods, e.g. pGenTHREADER [30], can be applied for transmembrane fold recognition with success. HHalign and Jackhammer use HMM approach to detect sequence relatives, while TMFR applies special scoring functions to align sequences and to predict, if the given sequence pairs share the same fold. pGenTHREADER applies statistical potentials and a neural network to find the native structure and estimate the confidence of the calculation.

HHalign, Jackhammer and pGenTHREADER are generic methods for structure homologue search and prediction. They do not integrate topography or topology to make predictions more accurate for TMPs. Furthermore, HHalign, Jackhammer and TMFR are not based on physical models which would open doors for understanding the details of structure formation and stability. Another disadvantage of them is that they are not able to take into account the environment of TMPs, e.g. contacts with other chains in an oligomer form or contacts with the lipid molecules.

Here, we present a statistical potential and a basic threading algorithm-based method, called TMFoldRec, which is able to predict the fold class of a given protein chain with 77 % accuracy. Moreover, it is shown that TMFoldRec distinguishes correctly the native and non-native structures with an accuracy of 70 % for the highest 40 % of the reliability values. TMFoldRec is tested on a dataset of 124 TMP structures.

## Implementation

The program for fold recognition and deciphering statistical potential has been written in C programming language. For collecting data, classifying structures Perl and Python scripts were written.

The pairwise structure and sequence alignments are stored in an appropriate MySQL database to avoid generating numerous files and to speed up searches for the computations.

## Methods

### Derivation of data set

For deriving a representative structure data set, structures determined by X-ray diffraction with a resolution better than 3 Å and annotated fully in the TOPDB database [31, 32] were selected from the redundant α-helical TMP set of the PDBTM database (revision 543) [33–35]. Then, TMP structures were disassembled into single chains. Chains with more than one membrane regions were selected and grouped based on the number of their membrane regions and the non-membrane regions were cut out from the structures. The side information of the soluble parts (hereinafter referred as topology) was taken from the PDBTM database.

All-versus-all structural alignments have been performed by TM-align algorithm [36] for all chain structures with same number of membrane regions. Based on the average TM-scores (provided by TM-align), chains were grouped. Structure pairs with an average TM-score greater than a threshold of 0.6 were assigned to the same fold class. Each class was filtered further for a sequence identity lower than 0.25 in the membrane region derived from the structure alignment. In this way, a representative data set (*R*) was created which is uniformly sampling the structure and sequence space.

The final set contains 142 TMP chains (see Additional file 1: Table S1 for PDB identifiers). The fold classes generated by this algorithm are in a perfect agreement with CATH superfamilies [37] for TMP chains with more than 2 TM regions (data not shown), but they contain the newest structures as well, which were solved after the last update of the CATH database. We note that the comparison was performed only on those TMPs, which are already classified in CATH.

### Development of statistical potential

For the development of statistical potentials, all the selected 142 TMPs were used. However, in the 10-fold jackknife test, TMPs with 2 or more than 16 TM regions were excluded. TMP chains with only 2 TM regions (17 chains) were skipped, since they form only a few contacts and differ only in the distances and the relative tilt angles of helices. TMPs with more than 16 TM regions (1 chain) were taken out from the set in order to decrease computational time. Altogether, 124 TMPs were used to evaluate the accuracy of our method.

In order to estimate the interaction energies between the amino acid pairs and that between the amino acids and their lipid ambient, a modified ENERGI algorithm was used [38]. This maximum likelihood algorithm, generating alternative structures (decoy or non-native) by random shuffling, maximizes the probabilities of the native structures over the alternative ones. *Θ* contains all the interaction parameters of the possible standard amino acid pairs and amino acid – lipid interactions. Residues are defined to be in contact if the distance of their C_β_ atoms (and C_α_ for glycine) is less than 5 Å and the sequence separation is larger than 4 residues. A residue is in contact with the lipid ambient if a heavy atom of the residue is accessible with a probe sphere of 1.4 Å from the lipid accessible protein surface. The calculation was performed in the same way as in the TMDET algorithm [35].

The energy function for a single sequence can be written as:

1 $$ {E}_{SEQ}\left({c}^{prot},{s}^{prot},,,\theta \right)={\displaystyle \sum_{i=0}^N{\displaystyle \sum_{j=i}^N{\varepsilon}_{ij}{n}_{ij}}+{\displaystyle \sum_{i=0}^N{h}_i{m}_i}}, $$

where *c* ^prot^ and *s* ^prot^ are the conformation and the sequence of a given protein chain, respectively, N = 20 for the standard amino acids, *ε*_ij_ and *h*_i_ are the elements of *Θ* and denote the interaction energy between the *i* ^th^ and the *j* ^th^ type of amino acids, which are closer than a cut-off value, and the interaction energy between the *i* ^th^ type amino acid and a lipid, respectively. In addition, *n*_ij_ and *m*_i_ are the number of contacts formed by *i* ^th^ and *j* ^th^ type amino acids and *i* ^th^ type amino acid with the lipid, respectively. For multiple sequence alignment Eq. 1 can be rewritten as

2 $$ {E}_{MSA}\left({c}^{prot},{s}^{MSA},,,\theta \right)=\frac{1}{M}{\displaystyle \sum_{k=1}^M{E}_{SEQ}\left({c}^{prot},{s}_k^{prot},\theta \right)}, $$

where *s* ^MSA^ is an array of sequences and M is the number of sequences in the multiple sequence alignment. For generating multiple sequence alignments, PSI-BLAST [39] was used on the UniRef50 database (release 2014–02) [40] with an iteration number of 3 and an E-value cutoff of 10^−5^. The optimization was performed by using a simple steepest descent method.

For a more detailed description of the statistical potential and its development, the reader is referred to Additional file 1.

### Mimic surrounding chains with a *z*-coordinate dependent amino acid distribution

To consider the surrounding chains in the cases of hetero-oligomer structures, a *z*-coordinate dependent average amino acid distribution was applied. It was determined by analyzing the representatively selected TMP structure set, *R*, as follows. The membranes were parallel to the *xy* plane and their core was at *z* = 0. The space was sliced parallel to the *xy* plane from *z* = 0 to |*z*| = 15 Å to 1 Å-thick-layers and for every slice, an overall amino acid distribution was calculated based on |*z*_*Cβ*_| values, the *z*-coordinate of the C_β_ atoms (or C_α_ for glycine). Then the normalized distributions were used to refill the missing information on the amino acids of a surrounding TM helix (in the case of hetero-oligomers) by taking the amino acid frequencies at |*z*_*Cβ*_| (see Additional file 1).

To process TMP structures and PDB files the in-house developed PdbLib was used, as in our previous works [33–35, 41].

### Prediction of the reliability of nativeness

To estimate the reliability of the fold predictions the following analysis was performed on the native and the lowest energy decoy structures. We defined the reduced energy, *e* = *E/NTM*, where *E* is the calculated energy of a given structure and *NTM* is the number of TM regions. The measured cumulative reduced energy distributions of the native and decoy structures were fitted with the following Gauss error function: *H* = erf(e*a-b)/2 + 0.5. The fit parameters, a and b, were found to be: a_NATIVE_ = 1.380, b_NATIVE_ = −2.473, a_DECOY_ = 2.050, b_DECOY_ = −3.145 for native and decoy structures, respectively (see Fig. 4). Using these fitted curves, the reliability of nativeness can be calculated as *H*_NATIVE_/(*H*_NATIVE_ + *H*_DECOY_). This score is between 0.5 and 1.

### Topology filtering

The topology of a given TMP sequence describes which regions are localized in the cytosolic, extra-cytosolic side or in the membrane slab. In this sense, every TMP has a grammatical structure, which can be also used to filter out incompatible folds. For instance, if a query sequence does not have any re-entrant regions, structure templates with re-entrant region(s) can be ignored. In the present work, topology information for the structural templates is taken from the PDBTM database and for the query sequences the topology was predicted by the CCTOP method (http://cctop.enzim.ttk.mta.hu).

The grammatical structure of a transmembrane sequence can be derived from topology information by concatenating the consecutive region types. Thus, topology information can be converted into a grammatical structure. (For instance, 11111MMMMM22222MMMMM22222MMMMM11111, where M, 1 and 2 denote the membrane region and its side one and side two, respectively, can be converted to 1M2M2M1.)

For topology filtering, the grammatical structure of the query sequence and that of the template are aligned. The goal of the analysis is to check the consistency of the side changes. A template is accepted if the similarity value (*TS*) is greater than 0.9. Since side 1 and side 2 are set by arbitrary in the PDBTM database, *TS* can be calculated as follows:

3 $$ TS=\frac{ \max \left({N}_{11}+{N}_{22},{N}_{12}+{N}_{21}\right)}{L-{N}_M}, $$

where *N*_11_ and *N*_22_ are the number of matching, *N*_12_ and *N*_21_ are the number of opposite localization of TMP sequence parts. *N*_M_ denotes the number of membrane regions and *L* is the length of a grammatical structure.

### Threading algorithm

A basic gapless threading method was utilized to search for the most likely structure. The membrane parts of the query sequence were aligned to the filtered template structures. In the procedure of the sequence-to-structure alignment, the membrane region of the sequence and that of the membrane-embedded structure parts were aligned with a shift from (*L*_str_i_-*L*_qry_i_)/2-2 to (*L*_str_i_-*L*_qry_i_)/2 + 2, where *L*_str_i_ and *L*_qry_i_ denote the length of the *i* ^th^ membrane region of the template structure and the query sequence, respectively. This resulted in 5^*NTM*^ alignments, where *NTM* denotes the number of transmembrane regions. In order to get rid of void positions in the structure, a 10-residue-long upstream and downstream region were appended to the membrane parts of the sequence.

## Results and discussion

### The TMFoldRec algorithm

TMFoldRec is a novel method to determine the fold class of TMPs. In addition to the sequence of TMPs, input parameters are the predicted or experimentally determined topologies of TMPs, as well. A statistical potential, which describes the energy contribution of amino acids and lipid contacts, is used to select the most likely structure as well as to predict the reliability of nativeness. For the energy calculation, the 20 standard amino acids are distinguished, but the lipid membrane is handled as a homogenous, continuous environment. Each interaction type is determined in a collective maximum likelihood procedure.

**Fig. 1:**
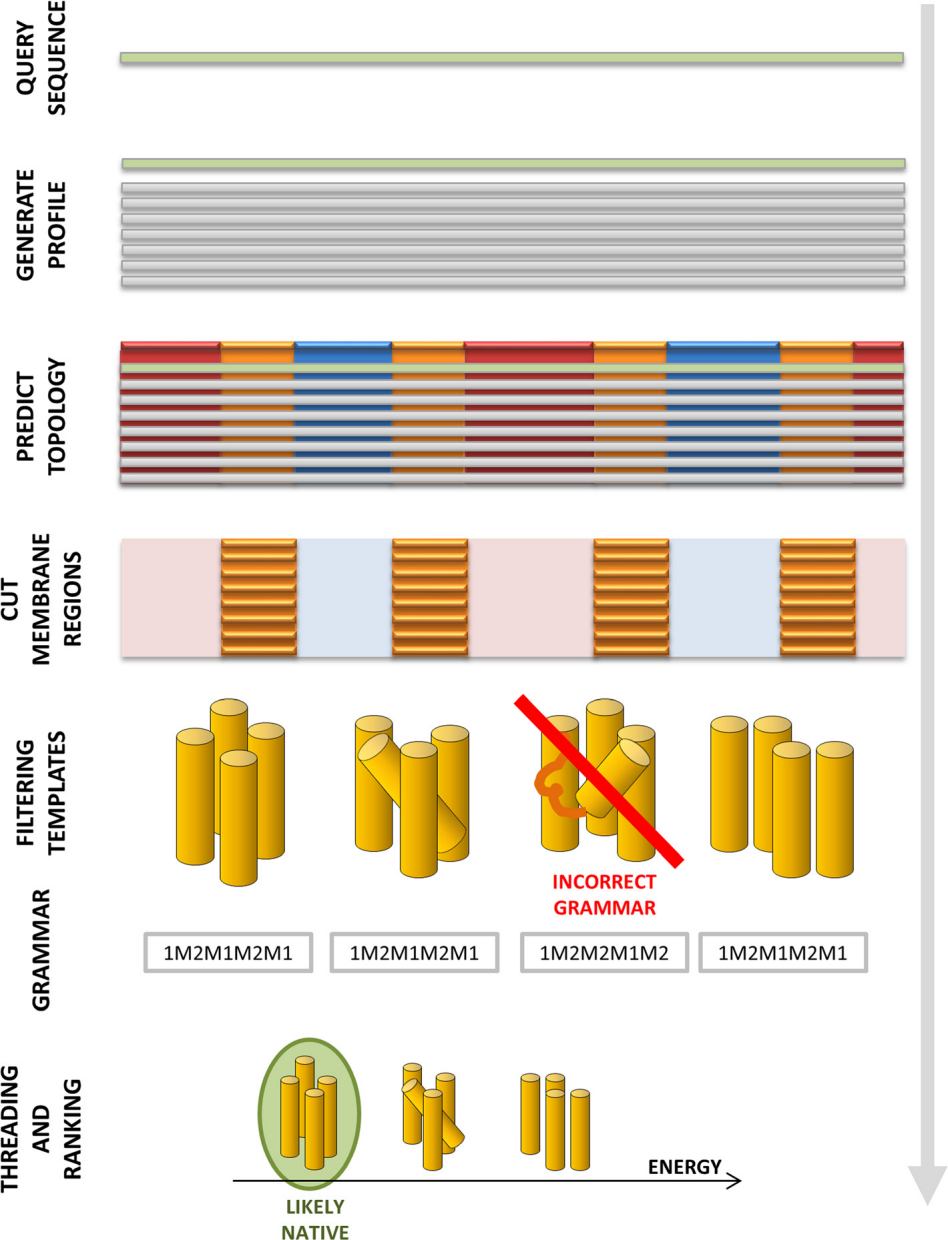
The main steps of TMFoldRec. The consecutive steps of TMFoldRec algorithm are the profile generation, topology prediction, extraction of membrane regions, filtering of template structures and threading.

The main steps of TMFoldRec are depicted in Fig. 1. For a query sequence, a multiple sequence alignment is generated by PSI-BLAST [39]. For developing statistical potential, the topology information defined in PDBTM database were used, while for measuring the accuracy of the method in the benchmark test, the topologies were predicted by a recently developed consensus constrained prediction method (CCTOP, http://cctop.enzim.ttk.mta.hu). Corresponding to the topology of the query sequence, TMFoldRec selects TMP structures with the same number of membrane regions from the previously collected representative structure database. Structures in this representative database contain the full membrane-embedded quaternary structure of the TMPs. Therefore, TMFoldRec takes different oligomeric forms and environments of TMPs into account in order to determine the most likely native fold. The knowledge of the surrounding molecules has a very important effect on the energy calculation and hence on the correct ranking of the various template structures, as well. As a consequence, using a single representative set of TMP chains for training and testing would abolish the information on inter-chain contacts and lipid accessibility. In order to avoid this bias, the effect of the surrounding chains is also taken into account. For homo-oligomer structures a periodic boundary condition corresponding to the symmetry of the biomolecule is applied to determine inter-chain contacts. For hetero-oligomer structures, the neighboring chains are modeled by a *z*-coordinate dependent average amino acid distribution (see Methods).

The TM parts of the query sequence profile are aligned systematically onto the structures with the same number of TM regions and the energy is calculated for each conformation. The structure with the lowest energy is selected as the most probable fold.

### Fold recognition of transmembrane proteins

**Fig. 2:**
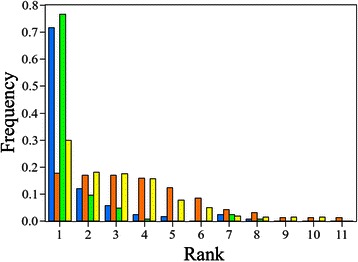
Performance of TMFoldRec. The performance of TMFoldRec algorithm was tested on 124 TMPs (2 < *NTM* ≤ 16). Blue bars denote the frequency of native folds ranked by TMFoldRec. Orange bars are the results of random prediction. Green bars denote the performance of TMFoldRec with topology filtering. The yellow bars are the corresponding random predictions.

TMFoldRec algorithm has been tested on a benchmark dataset with a 10-fold jackknife validation method. 90 % of the data was used to develop statistical potentials and the remaining 10 % were kept for the test of the potential set. In the 77 % of the cases, native folds were ranked at the first place, i.e., they had the lowest energy over the alternative folds (see Additional file 1). Using topology filtering only a slight performance increase (~5 %) could be achieved (Fig. 2).

For estimating the prediction baseline, the following method was applied, which is purely based on the number of the membrane embedded segments. If the fold library contains *F* different fold classes, where templates have as many membrane segments as the query sequence, the accuracy of the random predictor is 1/*F*.

**Fig. 3:**
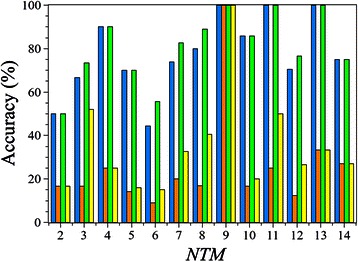
Performance of TMFoldRec in the function of *NTM*. Blue and green bars denote the topology filtered and non-filtered accuracy of TMFoldRec, respectively. The orange and yellow bars are the corresponding random predictions, which imply the number of different folds.

We have also checked, whether the performance of our algorithm depends on the number of transmembrane regions. Consequently, the prediction accuracy was plotted against the number of TMP regions. We found that the prediction accuracies were independent from the number of TM regions, i.e., no systematic tendency was observed (Fig. 3).

The weak performance on TMPs with 2 TM regions was probably caused by the small number of contacts formed between the membrane regions. In case of structures 4c9g_A, 3h90_A, and 2c3e_A (with 6 membrane regions), native structures were found with an unexpectedly high rank (above 6), which might be a hallmark of a wrong oligomerization state. The accuracy values for TMPs with 9 TM regions are 100 %, since there is only one fold class available (see Additional file 1). The fold class prediction for the sole TMP with 16 TM regions failed due to an erroneous topology prediction. Excluding these entries from the test set, the performance of TMFoldRec was still found to be 77 %.

According to the calculated potential matrix, contacts between charged amino acids are unfavorable in transmembrane proteins. In addition, residues with large side chain have lower pairing propensities due to the resulted reduced compactness of packing. Comparing to the potential set derived from globular proteins, presented in the original IUPred paper [42], the most striking difference is the lack of disulfide bridges. A slight increment can be observed in His-His interaction, which is usually bridged with a heme. The contact formation propensities of amino acid with polar uncharged side chains and glycine, proline are complementary for transmembrane and globular proteins. Lipid interaction energies in average are lower with an order of magnitude, which highlights dominant contribution of the amino acid – amino acid interactions in stability.

### Predicting the reliability of nativeness

Since only the one fifth of all TMP folds are known, a simple fold recognition algorithm on the current template structure set with an unknown TMP sequence would result in a high number of false positive results. To avoid this, the development of an additional measure is necessary, which detects, if the template set lacks the native fold of a query sequence. In our case, only residual contact information is available. Therefore, previously published structure assessing methods, such as ProQM [43], IQ [44] and QMEANBrane [45], cannot be used, because these methods require full atomic information besides the C_α_ or C_β_ coordinates. *Z-score* values [46, 47] did not result in a sufficient solution to discriminate decoy and native structures, let alone it hardly depends on the number of templates.

**Fig. 4:**
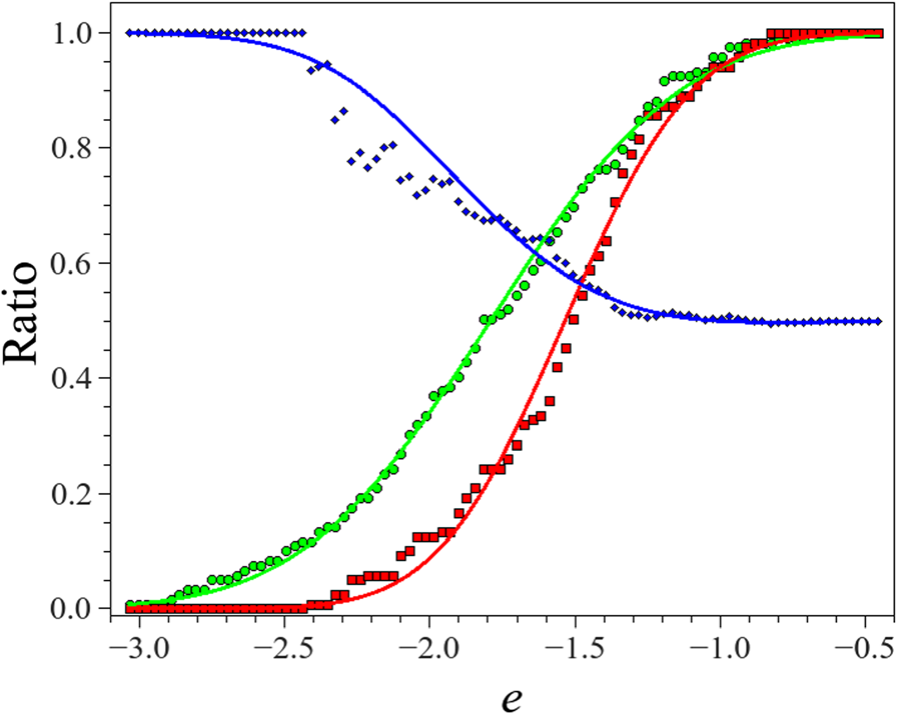
Performance of reliability prediction. Green and red dots denote the normalized cumulative histogram of reduced energy (*e*) values of native and decoy structures, respectively. The continuous green and red lines are the fitted curves. The blue dots are the calculated ratio of the native folds below the given reduced energy value. The blue line is the theoretical curve.

Based on the reliability of nativeness scores, TMFoldRec can effectively discriminate native folds from non-native ones with a remarkable confidence. As it can be seen in Fig. 4, for the highest 20 % of reliabilities, the accuracy is 80 %. For the 40 %, it is still 70 %. The most important property of this measure is that it is an absolute value and it does not depend on the number of templates, which is a great advantage over the ensemble-based measures (e. g. *Z*-score).

### Comparison with other methods

In order to compare the performance of TMFoldRec with the state-of-the-art methods, we benchmarked HHalign [17, 18], Jackhammer [48], RaptorX [49–51] and pGenTHREADER [30]. TMFR [29] was not available (nor upon request) during the development of TMFoldRec. Consequently, it has to be excluded from the comparison. These methods take the whole sequence to predict structural homology. In order to stave off an unfair assistance of domains in the soluble parts, Pfam annotated domain regions [52, 53] were cut out from the sequences if they do not overlap with the membrane segments. However, some soluble parts remained in the sequence, which provided additional information for these alignment-based methods. In the interest of correct testing of HHalign and Jackhammer, the same multiple sequence alignments were used as for TMFoldRec. However, RaptorX and pGenTHREADER was tested with their original template set (15/02/2015 and 05/01/2015, respectively). The predictions of these methods were filtered for TMPs with sequence identity of less than 40 % for a query sequence. If the TM-score between query and predicted template structure was greater than 0.6, predictions were taken as correct ones. The results of the benchmark tests are summarized in Table 1.

**Table 1:** Benchmark test of TMFoldRec and other fold predictors

Method	Accuracy
Jackhammer	43 %
HHalign	54 %
RaptorX	54 %
pGenTHREADER	63 %
TMFoldRec	77 %

As it can be seen in Table 1, TMFoldRec overcomes the current methods in TMP fold class prediction. However, we have to note that our representative data set contains clusters with only one chain and in these cases the alignment-based methods fail per definition, leading to a performance decrease. This highlights the advantage of structure-based fold recognition over alignment-based methods.

Excluding the one-element clusters and filtering out the sequence homologues of sequence identity greater than 25 % from the benchmark set only 26 chains remain. On this subset, the performance of the alignment-based methods increased, while that of TMFoldRec was found to be the unchanged. The accuracy of Jackhammer and HHalign methods were 58 % and 88 %, respectively. In this particular case, i.e., on the 21 % of the whole data set, HHalign was superior. However, it should not be forgotten that our method does not depend on the number of the known sequences and it has the ability to score structures and in this way to detect ambiguous structures (such like some of the 6-TM-chains).

## Conclusions

In this article, we have described a newly developed fold recognition algorithm, TMFoldRec for TMPs. The algorithm was tested on a set of 124 TMPs and a fold class prediction accuracy of 77 % was achieved. The advantages of TMFoldRec over the common methods were also presented. In contrast to the alignment-based methods, our algorithm has the ability to find the correct fold class even if no sequence homologue exists. Furthermore, instead of using machine learning approaches of HMM-based algorithms, TMFoldRec applies a strictly physical model with a minimal parameter set. Moreover, this physical model, which captures the main effects of amino acid interactions in the membrane regions, was found to be a proper basis for further considerations on the structure stability of TMP structures, as well. It was also presented that the native folds of various TMP sequences can be efficiently recognized with our statistical potential set. It implies, the structures of TM parts of TMPs are mainly the consequence of the favorable interactions of the spatially close residues in the TM region, and the constraints imposed by the water soluble parts of the same protein only perturb it. Currently our algorithm handle TM segments as independent parts and does not apply any geometrical constraints on the linker regions. We’ve tried to incorporate the information on linker length into the topology filtering by asymmetrically scoring alignments including short and long loops. When a short query linker (not longer than 5 residues) region aligned to a long template loop, template was rejected. Despite of the implementation of this additional filter the prediction accuracy remained the same, which could be the consequence of wrong partitioning of short and long loops as well as it could reconfirm the statement in the previous paragraph. A key feature of the method is its ability to predict the reliability, i.e., whether the predicted lowest energy fold represents the native structure. This information is crucial in case of TMPs when not all of the folds are known. This feature makes TMFoldRec a powerful tool for large scale transmembrane proteome scanning as well as for the determination of TMPs with unknown fold to reveal the structure space of TMP membrane domains more rapidly.

### Availability of supporting data

Mathematical details of optimization, the energy function and its calculation as well as the list of used TMP chains are included within the article and its additional file.

## Additional file

Additional file 1:
**Mathematical details of optimization, the energy function, the derived statistical potential and its calculation as well as the list of used TMP chains.**
